# Biological Responses of Human Gingival Fibroblasts (HGFs) in an Innovative Co-Culture Model with *Streptococcus mitis* to Thermosets Coated with a Silver Polysaccharide Antimicrobial System

**DOI:** 10.1371/journal.pone.0096520

**Published:** 2014-05-07

**Authors:** Silvia Sancilio, Viviana di Giacomo, Mara Di Giulio, Marialucia Gallorini, Eleonora Marsich, Andrea Travan, Lorena Tarusha, Luigina Cellini, Amelia Cataldi

**Affiliations:** 1 Department of Pharmacy, “G. d’Annunzio” University, Chieti-Pescara, Italy; 2 Medicine, Surgery and Health Sciences, University of Trieste, Trieste, Italy; 3 Department of Life Sciences, University of Trieste, Trieste, Italy; University Hospital of the Albert-Ludwigs-University Freiburg, Germany

## Abstract

This study sought to evaluate the *in vitro* biological response of human gingival fibroblasts (HGFs) co-coltured with *Streptococcus mitis* to bisphenol A glycidylmethacrylate/triethylene glycol dimethacrylate (BisGMA/TEGDMA) thermosets coated with Chitlac-nAg, a nanocomposite system with antimicrobial properties. To avoid bacterial adhesion to dental devices and to reduce cytotoxicity against eukaryotic cells, we coated BisGMA/TEGDMA methacrylic thermosets with a new material, Chitlac-nAg, formed by stabilizing silver nanoparticles, which have well-known antimicrobial properties, with a polyelectrolyte solution containing Chitlac. Cytotoxicity, cell morphology, cell migration and inflammatory interleukine-6 (IL-6) and prostaglandin E_2_ (PGE_2_) secretion were evaluated. Our results showed that the cytotoxicity exerted on HGFs by our nanocomposite material was absent in our co-culture model, where fibroblasts are able to adhere and migrate. After 24 h thermosets coated with Chitlac as well as those coated with Chitlac-nAg exerted a minimal cytotoxic effect on HGFs, while after 48 h LDH release rises up 20%. Moreover the presence of *S. mitis* reduced this release in a greater amount with Chitlac-nAg coated thermosets. The secretion of IL-6 was significant in both Chitlac and Chitlac-nAg coated thermosets, but PGE_2_ production was minimal, suggesting that the IL-6 production was not related to an inflammatory response. Co-culture and the addiction of saliva did not influence IL-6 and PGE_2 _secretion. Data obtained in the present work suggest that Chitlac n-Ag coated thermosets could significantly improve the success rates of restorative dentistry, since they limit bacterial adhesion and are not toxic to HGFs.

## Introduction

The increased use of composite materials in restorative dentistry has not been free of problems related to infections, the main reason for the failure of dental devices [1], [2]. The surfaces of the oral cavity are always exposed to a broad variety of microorganisms that colonize not only oral mucosa and teeth, but also the components used for restoration, leading to periodontitis and dental caries [3]. Recently, materials with antimicrobial properties have been proposed in order to avoid the proliferation and the adhesion of bacteria on their surface [4], [5]. Silver is well known for its antimicrobial properties and can be used in the form of nanoparticles, ions or salts in a variety of medical and general devices in order to retard and avoid bacterial infection [6], [7]. Silver ions and nanoparticles are capable of destroying the bacterial cell wall by reacting with electron donor groups, especially sulfhydryl groups on trans- and outer-membrane proteins, including proteins of the electron transport chain, protruding from the extracellular portion of the membrane [8]. Although eukaryotic cells lack such extracellular binding sites, they are able to internalize silver nanoparticles [9]; the diffusion of nanoparticles into the cytoplasm can lead to eukaryotic cell death by interfering with several metabolic pathways [10].

With the goal of preventing aggregation of silver nanoparticles, which can affect their antimicrobial activity, a lactose-modified chitosan was developed and has proven to be effective in stabilizing colloidal solutions of silver nanoparticles (“Chitlac-nAg”) [6], [9], [11]. Moreover, internalization of nanoparticles can be prevented by anchoring them into a stable and biocompatible polymeric film so that the nanoparticles interact directly with bacterial membrane without affecting eukaryotic cells [9]. In this work, we tested the efficacy of a Chitlac-nAg coating on a thermoset based on Bisphenol A glycidylmethacrylate (BisGMA) and triethylene glycol dimethacrylate (TEGDMA), a composition widely used for dental devices [12].

Even though the use of this coating for orthopaedic applications has already been examined through biological tests to evaluate its effects on both bacteria and eukaryotic cells [8], [9], [13], its use in the oral cavity involves a different, complex environment that includes eukaryotic gingival, epithelial and fibroblastic cells, human oral microbiota and saliva. This essential fluid maintains the oral ecosystems by ensuring the presence of water and nutrients, as well as adherence and antimicrobial factors. Saliva contains 99% water, enzymes, glycoproteins (including mucins), hormones, vitamins, urea and several ions [14]. The role of mucins in bacterial adherence is complex. When salivary glycoproteins are adsorbed on a solid surface, they may bind to bacteria and promote bacterial adherence. Instead, some of this glycoprotein, when free in saliva, may prevent bacterial colonization by binding to them or by agglutinating bacteria [15]. The human oral cavity is colonized by a crowd of microorganisms of which streptococci are the most plentiful. Commensal bacterial species can form tight associations with host epithelial tissue, benefitting oral health [16], [17]. *Streptococcus mitis*, a commensal microorganism, that is part of the oral flora, colonizes hard surfaces in the oral cavity such as dental hard tissues, as well as mucous membranes.

Although it is well known that resin composites like HEMA and TEGDMA induce inflammation and oxidative stress in HGFs [18]–[20], recent findings have demonstrated that *S. mitis* and saliva could mitigate the cytotoxic effects exerted by HEMA (2-hydroxyethylmetachrylate) [21], [22]. Thus, the aim of this study was to investigate cytotoxicity, migration, morphology and inflammatory cytokine production of HGFs grown on BisGMA-TEGDMA thermosets coated with Chitlac-nAg and co-cultured with *S. mitis* in the presence of saliva [23], in order to clarify the biological reactions occurring between biomaterials, host tissue and microbial environment.

## Materials and Methods

### Preparation of BisGMA/TEGDMA Thermosets (Uncoated Samples)

BisGMA (70% w/w) and TEGDMA (30% w/w) were mixed under vigorous stirring at 37°C. Camphorquinone (CQ, 0.7% w/w) and 2-dimethylamino ethylmethacrylate (DMAEMA, 0.7% w/w) were added and the solution was protected from light and degassed for 12 h in vacuum oven at 40°C. The solution was poured into a Teflon mold (diameter 14 mm, h 2.5 mm) and the wells were covered with a polyethylene terephthalate (PET) film. The polymerization was initiated with a halogen curing light (Optilux 501, λ: 400–505 nm, light power: 850 mW/cm^2^) for 20 s. The postcuring was performed with a Photopol IR/UV Plus oven (Dentalfarm, Torino, Italy) equipped with eight lamps and two spots operating in the wavelength range 320–550 nm following the procedure: 20 min in light oven (eight lamps), 20 min in light oven (eight lamps) on a rotating plate, 60 min in light oven (eight lamps) under vacuum, and 7 min in light oven (eight lamps plus two spots). The thermosets were then sandpaper polished (granulometry: 1200).

### Chitlac–nAg Preparation and Coating on the BisGMA/TEGDMA Thermosets

Silver nanoparticles were obtained by reducing silver ions with ascorbic acid in Chitlac solutions according to the procedure already described [8]. Briefly, Chitlac was dissolved in deionized water at a concentration of 4 g l^−1^. The Chitlac solution was mixed with AgNO_3_ solution to final AgNO3 concentrations of 1 mM and 2 mM. Ascorbic acid (C_6_H_8_O_6_) was added at final concentrations of 0.5 mM and 1 mM, respectively. After 4 h, a yellow–orange stable colloidal solution was obtained. BisGMA/TEGDMA thermosets were prepared and coated with Chitlac–nAg, as already reported [9]. Chitlac or Chitlac–nAg coating of the thermosets were obtained after surface activation with COO^−^ functional groups by hydrolysis of the methacrylate esters. The samples were immersed in HCl 12 M for 7 h at 80°C, rinsed alternately with deionized water and NaOH 0.1 M and finally air dried. The activated samples were immersed for 24 h in Chitlac or Chitlac–nAg solution and subsequently rinsed in deionized water for 1 h under agitation. The samples were dried under a hood and both sides of the thermosets were sterilized by 1 hour-cycle under UV light.

### Culture of Human Gingival Fibroblasts

Human gingival fibroblasts (HGFs) were obtained from fragments of healthy marginal gingival tissue taken from the retromolar area withdrawn during surgical extraction of impacted third molars in adult subjects following regularization of the surgical flap before sutures. Signed informed consent was obtained from the donors. None of the authors participated to the sample collection and samples were anonymized before the authors received them. The tissue fragments were immediately placed in Dulbecco’s modified Eagle’s medium (DMEM, EuroCloneSpA Life-Sciences-Division, Milano, Italy) for at least 1 h, rinsed three times in phosphate-buffered saline solution (PBS, EuroCloneSpA), minced into small tissue pieces, and cultured in DMEM, containing 10% foetal bovine serum (FBS), 1% penicillin, 1% streptomycin, and 1% fungizone (Sigma-Aldrich, Milan, Italy). Cells were maintained at 37°C in a humidified atmosphere of 5% (v/v) CO_2_. After one week, the fungizone was removed from the culture medium. Cells were used after 4–8 passages.

### Bacterial Strains and Growth Condition

The clinical strain *Streptococcus mitis* DS12 from a saliva sample was used in the present study. The strain was cultured in Trypticase soy broth (TSB, Oxoid, Milan, Italy) at 37°C for 18–24 h under anaerobic atmosphere. The overnight culture was diluted 1∶10 (v/v) in antibiotic and serum-free DMEM plus 1% (w/v) sucrose and refreshed for 2 h at 37°C in an orbital shaker at 160 rpm in aerobic condition; subsequently, the broth culture was adjusted to 0.5 McFarland in the same medium and used for the co-culture assays.

### Saliva Collection

Pooled unstimulated saliva was obtained from healthy laboratory staff, who having refrained from drinking and eating the previous two hours, spat samples into a polypropylene tube. The volunteers do not smoke and are not subjected to drug treatments. The saliva was then slowly stirred for 10 min and subsequently clarified by centrifugation at 16,000 g for 1 h at 4°C to remove debris, sterilized through a 0.2 µm filter and frozen at −20°C and processed within two days for the co-culture and for the aggregation test.

The sterility of the saliva was verified by incubating a small portion in TSB medium, in aerobic and anaerobic atmosphere for 24–48 h at 37°C.

### Co-culture Assay [24]

The co-culture assay was performed in cell culture flasks (Nunc, EuroCloneSpA). When HGFs reached confluence, the culture medium was removed and the standardized bacterial cultures in DMEM 1% sucrose were then added. When indicated, saliva (10%) and/or thermosets (placed onto the cell layer surface directly) were added. The samples were incubated for 24 and 48 h in a humidified atmosphere of 5% (v/v) CO_2_ at 37°C. The experimental design was carried out for at least three independent experiments.

The following experimental conditions were used:

**U** Human gingival fibroblasts (HGFs)

**Th** HGFs in the presence of Chitlac coated thermosets

**Ag** HGFs in the presence of Chitlac-nAg coated thermosets

**MTh** HGFs in the presence of *S. mitis* and Chitlac coated thermosets

**MAg** HGFs in the presence of *S. mitis* and Chitlac-nAg coated thermosets

**MSTh** HGFs in the presence of *S. mitis*, saliva and Chitlac coated thermosets

**MSAg** HGFs in the presence of *S. mitis*, saliva and Chitlac-nAg coated thermosets

After incubation, cells were washed with PBS, trypsinized, and processed for the Trypan Blue dye exclusion test, which selectively identifies dead fibroblasts in blue.

### Cytotoxicity (LDH) Assay

HGFs were seeded at 200,000 cells/well on 24 wells plates. After 24 h, the culture medium was replaced with 1 ml of DMEM 1% sucrose and thermosets were placed directly on the cell layer. Where indicated, the standardized bacterial cultures were added (final volume 1 ml). After 24 and 48 h, the medium was harvested and lactate dehydrogenase based assay (LDH assay, TOX-7, Sigma-Aldrich, St. Louis, MO) was performed on the culture media according to the manufacturer’s instructions. As positive control, cells were lysed with Triton 1%. Each test was performed in quadruplicate. Assessment of cytotoxicity was calculated according to the formula: %LDH released = [(A–B)/(C–B)] ×100, with A = LDH activity of sample, B = LDH activity of untreated cells and C = LDH activity of the positive control.

### Transwell Migration

The quantitative migration assay was performed using a modified Boyden chamber (Transwell, Corning, NY, USA) with 8.0-µm pore polycarbonate filter inserted in a 24-well plate. In the top chamber, HGFs were present in different culture conditions. The bottom chamber was filled with 600 µl of DMEM 0.1% sucrose. Thermosets were always placed in the bottom chamber while, unless otherwise indicated, saliva and bacteria were added to the cells in the top chamber. Following the incubation (24 h), migrated cells were trypsinized, harvested and then resuspended in complete medium. The number of migrated cells was assessed by flow cytometry by counting the cells flowing in a definite acquisition time (30″) and in a known volume (150 µl).

### Optical Microscopy

HGFs were grown on 24-well culture plates and allowed to adhere. After 24 h, the medium was replaced with 1 ml of DMEM 1% sucrose, then each thermoset (ø = 14 mm; h = 2.5 mm) was cut into four equal parts and one part was placed in each well on the cells. When called for, standardized bacterial culture was added. After 24 and 48 h, the observation was carried out using a phase-contrast light microscope (LEICA, Wetzlar, Germany) equipped with a CoolSNAP videocamera for acquiring computerized images (Photometrics, Tucson, AZ).

### Enzyme-Linked Immunosorbent Assays (ELISA)

HGFs were seeded at 200,000 cells/well on 24 wells plates. After 24 h, culture medium was replaced with 1 ml of DMEM 1% sucrose and thermosets were directly placed on the cell layer. Where called for, the standardized bacterial cultures were added (final volume 1 ml). After 48 h, medium from each well was harvested and secretion of interleukine-6 (IL-6) and prostaglandins E_2_ (PGE_2_) in the culture media was evaluated by ELISA kit (both from Enzo Life Sciences, Farmingdale, NY, USA), according to the manufacturer’s instructions.

Absorbance values were obtained by reading at 450 and 405 nm (for IL-6 and PGE_2_, respectively) using an Anthos 2010 microtest plate spectrophotometer (Anthos Labtec Instruments, Salzburg, Austria).

### Statistics

Statistical analysis was performed using the analysis of variance (ANOVA). Results were expressed as mean ± SD. Values of p<0.05 were considered statistically significant.

## Results

### LDH Cytotoxicity Assay

**Figure 1:**
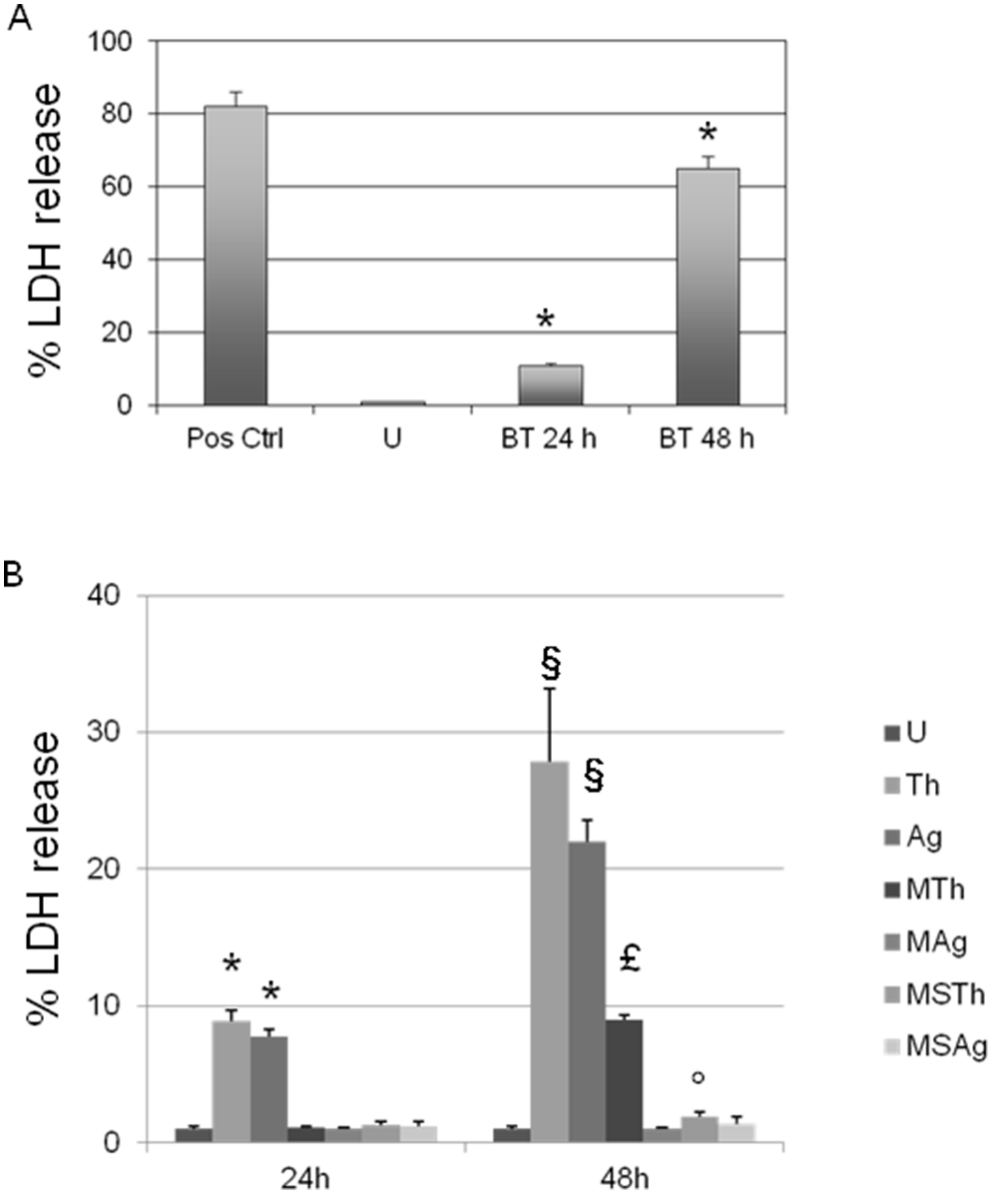
Effect of BisGMA/TEGDMA thermosets on LDH release. **A**: in HGFs. The graph represents the mean percentage ± SD of three different consistent experiments. *BT 24 h and BT 48 h vs U, p = 0.0392 and p = 0.0019. **B**: in HGFs/*Streptococcus mitis* co-culture model. The graph represents the mean percentage ± SD of three different consistent experiments. **U**: HGFs; **BT**: HGFs with uncoated thermoset; **Th**: HGFs with Chitlac thermoset; **Ag**: HGFs with Chitlac-nAg thermoset; **MTh**: HGFs with Chitlac thermoset and *S. mitis*; **MAg**: HGFs with Chitlac-nAg thermoset and *S. mitis*; **MSTh**: HGFs with Chitlac thermoset, *S. mitis* and saliva; **MSAg**: HGFs with Chitlac-nAg thermoset, *S. mitis* and saliva. *Th and Ag 24 h vs U 24 h, p = 0.0335 and p = 0.0018; § Th and Ag 48 h vs U 48 h, p = 0.0257 and p = 0.0181; £ MTh 48 h vs Th 48 h, p = 0.0304; °MSTh 48 h vs MTh 48 h, p = 0.0272.

Uncoated BisGMA/TEGDMA (BT) thermosets placed on HGFs showed time dependent cytotoxicity, compared to the lactate dehydrogenase (LDH) kit positive control (Fig. 1A). In fact, the percentage of LDH released was 11 and 65 fold higher at 24 and 48 h, respectively compared to the untreated sample (indicated in tables and figures as U).

Figure 1B shows the percentage of LDH in the different experimental conditions. After 24 h thermosets coated with Chitlac as well as those coated with Chitlac-nAg exerted only a minimal cytotoxic effect on HGFs (8.9±0.8 and 7.8±0.5%, respectively), while LDH release at 48 h rises up to 27.9±5.3 and 22.0±1.6%. Moreover, in the presence of *S. mitis*, a greater reduction of LDH release by HGFs was seen with the Chitlac-nAg coated thermosets (indicated in figure as MAg, 1.0±0.1%) than with those coated with Chitlac alone (indicated as MTh, 9.0±0.3%) coated thermosets.

When saliva was added to each experimental point, LDH release returned to basal levels after 24 and 48 h of treatment.

### Migration Assay

**Figure 2:**
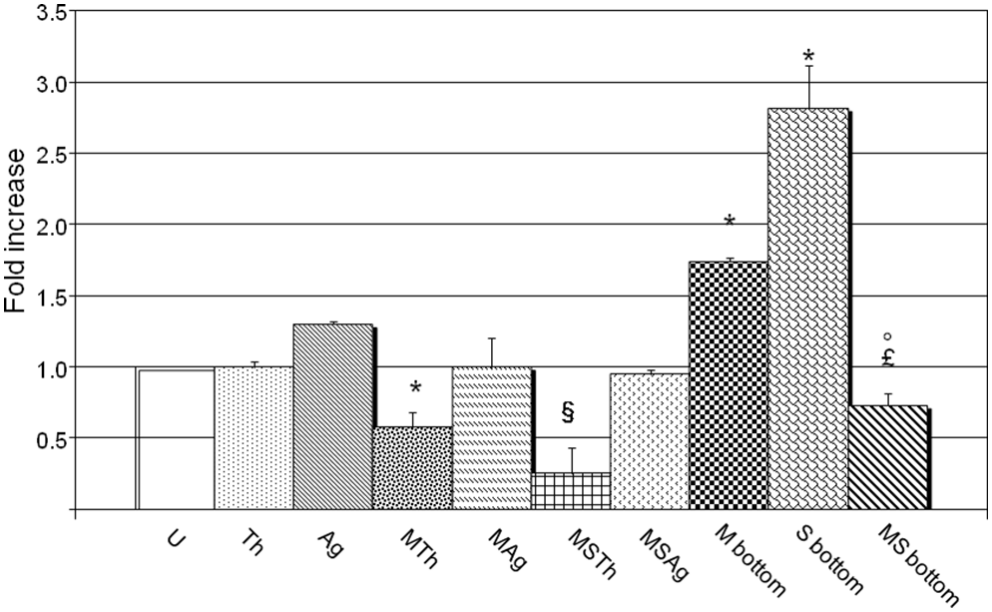
Effect of Chitlac and Chitlac-nAg thermosets on cell migration in HGFs/*Streptococcus mitis* co-culture model. The graph represents the mean fold increase of cell number ± SD of three experiments. **U**: HGFs; **Th**: HGFs with Chitlac thermoset; **Ag**: HGFs with Chitlac-nAg thermoset; **MTh**: HGFs with Chitlac thermoset and *S. mitis*; **MAg**: HGFs with Chitlac-nAg thermoset and *S. mitis*; **MSTh**: HGFs with Chitlac thermoset, *S. mitis* and saliva; **MSAg**: HGFs with Chitlac-nAg thermoset, *S. mitis* and saliva; **M bottom**: *S. mitis* in the bottom chamber: **S bottom**: Saliva in the bottom chamber; **MS bottom**: *S. mitis* and saliva in the bottom chamber. *MTh, M bottom, S bottom vs U, p = 0.0421, p = 0.0397, p = 0.0041; § MSTh vs MTh, p = 0.0461; £ MS bottom vs M bottom, p = 0.0037; °MS bottom vs S bottom, p = 0.0234.

The first quantitative migration assay, using a modified Boyden chamber (Fig. 2), was performed at 24 h to evaluate the ability of the thermosets to attract the cells. Both Chitlac and Chitlac-nAg thermosets (indicate as Th and Ag) induced a cell migration similar to that of the control (U). The second migration assay was performed in presence of *S. mitis*. When a Chitlac thermoset was placed in the bottom chamber, and *S. mitis* in the top chamber (*S. mitis* + Chitlac is indicated as MTh in the figures) cell migration was reduced (0.58±0.10 p = 0.0421); instead, when a Chitlac-nAg thermoset was placed in the bottom chamber, and *S. mitis* in the top chamber (*S. mitis* + Chitlac-nAg is indicated as MAg in the figures MAg) the number of migrated cells was not affected remaining comparable to that of the control (1±0.20).

In the third migration assay, saliva was added to the co-culture in the top chamber. When a Chitlac-coated thermoset was placed in the bottom chamber (*S. mitis* + saliva + Chitlac is indicated as MSTh in figures) there was a further decrease in cell migration MSTh 0.25±0.18 *vs* MTh p = 0.0461) while no significant changes were observed when the thermoset in the bottom chamber was coated with Chitlac-nAg (*S. mitis* + saliva + Chitlac-nAg is indicated as MSAg in figures) (MSAg 0.95±0.03 *vs* MAg).

A fourth set of migration assays was conducted to better assess the capability of *S. mitis* to attract HGFs. When bacteria were placed in the bottom chamber (M bottom), a considerable increase in cell migration was observed (1.74±0.02 p = 0.03972). The addition of saliva to bacteria in the bottom chamber (MS bottom) caused a drastic decrease in cell migration (MS bottom 0.73±0.08 *vs* M bottom p = 0.0037) while saliva alone increased cell migration (S bottom 2.13±0.30 *vs* MS bottom p = 0.0234).

### Light Microscopy

**Figure 3:**
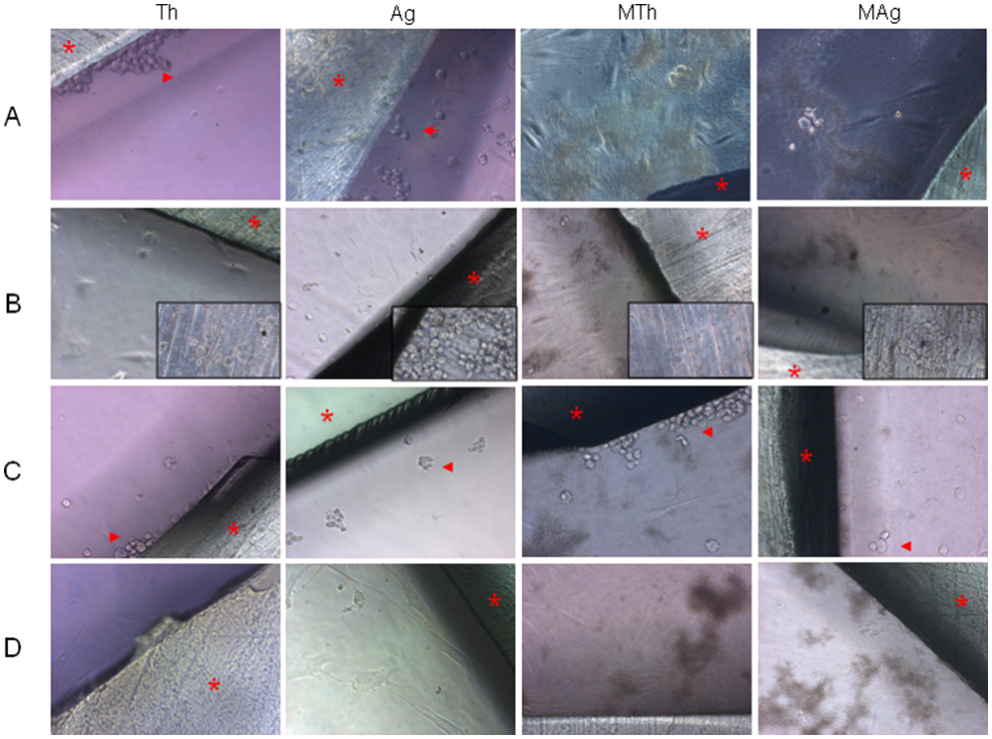
Effect of Chitlac and Chitlac-nAg thermosets on cell morphology in HGFs/*Streptococcus mitis* co-culture model. Magnification 20X **A)** 24 hours treated samples **B)** 48 h treated samples. Insets show cells on thermosets surface **C)** 24 h saliva treated samples **D)** 48 h saliva treated samples. Asterisks indicate thermoset surface; arrows heads indicate detached cells. **Th**: HGFs with Chitlac thermoset; **Ag**: HGFs with Chitlac-nAg thermoset; **MTh**: HGFs with Chitlac thermoset and *S. mitis*; **MAg**: HGFs with Chitlac-nAg thermoset and *S. mitis.*

In order to assess the interaction between cells and Chitlac or Chitlac-nAg thermosets, samples were observed using light microscopy. At 24 h, light phase-contrast pictures revealed that HGFs detached from flask bottom when in contact with thermoset coated with Chitlac as well as with those coated with Chitlac-nAg. When *S. mitis* was added to the culture, cells remained adherent to the plate (Fig. 3A). Unexpectedly, after 48 h HGFs cultured in absence of bacteria appeared thin, elongated and adherent while no changes were observed in the presence of bacteria (Fig. 3B). Moreover, after 48 h of incubation, round-shaped cells were observed on thermoset surface (Fig. 3B, insets).

After 24 h in the presence of saliva, HGFs also detached in the co-culture condition albeit to a minor extent with respect to controls (Fig. 3C). At 48 h the cells adhered again in all the experimental conditions (Fig. 3D).

### IL-6 and PGE_2_ Release

**Figure 4:**
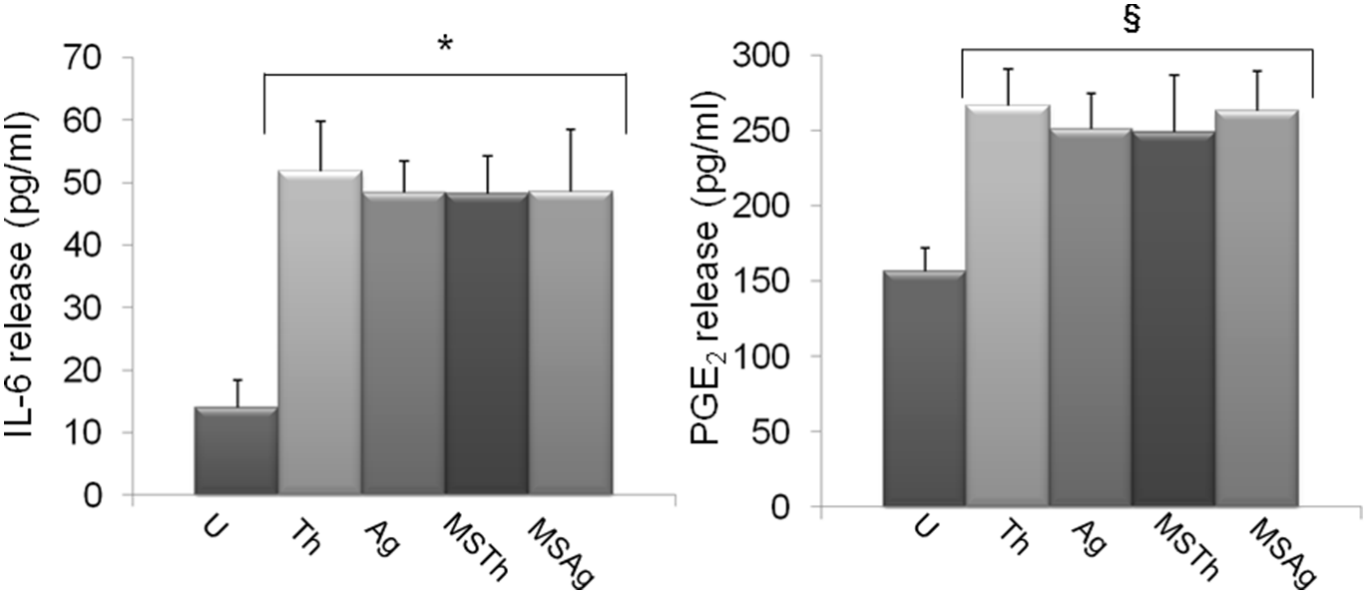
Effect of Chitlac and Chitlac-nAg thermosets on IL-6 and PGE_2_ release in HGFs/*Streptococcus mitis* co-culture model. Graph represents the mean concentration (pg/ml) ± SD of three different consistent experiments. **U**: HGFs; **Th**: HGFs with Chitlac thermoset; **Ag**: HGFs with Chitlac-nAg thermoset; **MSTh**: HGFs with Chitlac thermoset, *S. mitis* and saliva; **MSAg**: HGFs with Chitlac-nAg thermoset, *S. mitis* and saliva; *Th, Ag, MSTh and MSAg vs U IL-6, p = 0.0391, p = 0.0299, p = 0.0325, p = 0.0408; § Th, Ag, MSTh and MSAg vs U PGE_2_, p = 0.0300, p = 0.0262, p = 0.0472, p = 0.0362.

To evaluate the inflammatory response of HGFs to thermosets exposure, IL-6 and PGE_2_ release was measured (Fig. 4). Both Chitlac (Th) and Chitlac-nAg (Ag) coated thermosets showed an increase in release of IL-6 (51.73±8.01 and 48.36±5.10 pg/ml, respectively) and PGE_2_, (266.34±24.61 and 250.67±24.12 pg/ml, respectively) with respect to the untreated sample (U). Surprisingly, addition of saliva to the HGFs co-cultured with *S. mitis* did not influence IL-6 secretion and PGE_2_ production.

## Discussion

The cytotoxic effects on human gingival fibroblasts caused by biomaterials commonly used in restorative dentistry, have been the subject of considerable attention [25]. The antimicrobial efficacy and biocompatibility of the thermosets used in this study were also investigated previously in applications for orthopedic devices [8], [9], [26].

Since a previous study of ours examined the antibacterial and biofilm effects of Chitlac-nAg colloidal solution on oral streptococcal strains [11], in the present work we have focused on the effect of Chitlac and Chitlac-nAg thermosets on an innovative HGFs/*S. mitis*/saliva co-colture model which resembles the oral environment. In our experimental system, both kinds of thermosets exerted a time dependent toxicity on HGFs, as shown by LDH release assays. These results were confirmed by optical microscopy observation, which showed detached cells, especially when HGFs were in contact with thermosets.

When *S. mitis* and saliva were present, we observed a reduction in LDH release and in the number of detached cells, in line with previous studies [22]. As known, oral *Streptococci* have a membrane protein capable of interacting with eukaryotic cell membrane proteins, such as integrins [27], while saliva has a well known role in protecting cells from pathogenic microorganisms [22], [28]. Since saliva proteins can bind cells and influence their physiology [28], we hypothesized that in our experimental model the hydrophilic film created by saliva on the surface of HGFs strengthens cell interactions.

Moreover, *S. mitis* and saliva reduced cell migration rate towards Chitlac thermosets, while no change was observed when the thermoset was coated with Chitlac-nAg. This response can be explained by the fact that silver is toxic for bacteria [6], [11] and does not allow *S. mitis* to interact with HGFs. Di Giulio et al [11] demonstrated that nanocomposite system Chitlac-nAg, when not anchored on thermosets, is toxic both on sessile and planktonic phase of *S. mitis*. The binding to thermoset reduces the cytotoxicity on eukaryotic cells, without altering the toxicity on microorganisms [8].

It is interesting that HGFs were significantly attracted by the *S. mitis* and saliva placed in the bottom chamber: since eukaryotic cell interaction with microorganisms has been well studied [29] and the signaling pathway for integrin β 1, a protein in the membrane of HGFs, is modulated by *S. mitis* and saliva [21], we can hypothesize a kind of interaction and bonding reinforcement in our experimental system. When saliva and *S. mitis* were placed together in the bottom chamber, migration of HGFs from the top chamber was drastically reduced to a value lower than that of the untreated sample. This could be due to an interaction between the microorganism and saliva, through the amylase binding protein C (AbpC), produced by *S. mitis* [30], which in turn prevents both of them from interacting with HGFs.

Microscope observations showed the cytotoxic effects of the BisGMA-TEGDMA thermoset formulation on human gingival fibroblasts (cells appear detached) and the protective effect of saliva and *S. mitis*. In our experimental model, the release of IL-6 and PGE_2_ was not in line with that seen in previous studies that demonstrated an inflammatory response in HGFs exposed to different resin composites [17], [31]. HGFs are an important regulatory cell-type in the progression of periodontitis. Inflammation, a key event in the progression of periodontitis, is aggravated by host proinflammatory cytokines. Among them, the intensity of IL-6 expression is positively correlated with attachment loss [32]. Moreover, under single-species bacterial challenge or stimulation with inflammatory mediators, HGFs can produce high levels of PGE_2_, pleiotropic molecules whose production in periodontal disease is well known [33].

On the other hand, *S. mitis* has been shown to exert a strong immunomodulatory effect on human cells. The tissue destruction associated with periodontal disease is largely mediated by the host inflammatory response to infection by oral pathogens. On its own, *S. mitis* does not promote cytokines expression, being the co-incubation with other oral pathogens necessary to promote its transition from a commensal to a pathogenic state [34].

In the present work, IL-6 production was significant, although it showed no decrease in the presence of bacteria and saliva, while other parameters discussed above indicate improved cell health. Moreover, since PGE_2 _secretion was not striking either, it is possible that IL-6 production is not related to inflammatory response. Since it is already known that chitosan membranes can induce chondrogenic differentiation in HGFs [35], and that IL-6 can be involved in the activation of pathways different from the inflammatory one [36], and considering that in our experiments the cells that migrated to the thermosets were round, we can suggest that IL-6 plays a role in HGF differentiation induced by Chitlac coated thermosets. Further work is underway to support this hypothesis.

## Conclusion

All in all, our observations of the biological and molecular events provoked by treatment with Chitlac coated thermosets, in an *in vitro* in a co-culture model, that mimics the environment of the oral cavity, confirm the key role of oral bacteria and saliva in preventing toxic events that can occur *in vivo* in human gingival fibroblasts when BisGMA-TEGDMA is used for dental work, and lay the groundwork for further studies on novel roles for “old” molecules.
